# Intraosseous monitoring of drilling in lumbar vertebrae by ultrasound: An experimental feasibility study

**DOI:** 10.1371/journal.pone.0174545

**Published:** 2017-05-01

**Authors:** Nahum Rosenberg, Jacob Halevy-Politch

**Affiliations:** 1 The Ruth and Bruce Rappaport Faculty of Medicine, Technion–Israel Institute of Technology, Bat Galim, Haifa, Israel; 2 JetGuide Ltd., Haifa, Israel; Universita degli Studi di Palermo, ITALY

## Abstract

The rationale for this project is to evaluate the efficiency of a novel sonographic method for measurements of interosseous distances. The method utilizes a propagating ultrasonic beam through aqueous milieu which is directed as a jet into a drilled tract. We used a plastic model of human L5 vertebra and ex vivo specimen of L5 porcine vertebra and generated 2 mm in diameter tracts in vertebral pedicles. The tracts were created in the “desired” central direction and in the “wrong” medial and lateral directions. The drilled tracts and the residual, up to opposite cortex, distances were measured sonographically and mechanically and compared statistically. We show that "true” mechanical measurements can be predicted from sonographic measurements with correction of 1–3 mm. The correct central route can be distinguished from the wrong misplaced routes. By using the sonographic measurements, a correct direction of drilling in the pedicle of lumbar L5 vertebra can be efficiently monitored.

## Introduction

Surgical procedures for spinal stabilization or for gaining access into vertebral body are commonly utilize posterior approach and intra-pedicullar drilling. The intra-pedicullar drilling is a common surgical procedure that might be complicated by a devastating neural root damage, neural canal penetration or penetration of anterior cortex of vertebra. Although the incidence of false route generation is relatively low, around 3% of operations [1], the result of such mal-tracking might cause a serious disability or may be even life threatening. Therefore, effective monitoring of intraosseous intra-pedicullar drilling is of high importance.

Usually experienced surgeons recognize well the anatomy of the vertebra and direct the intra-pedicullar drilling accordingly. This procedure is also assisted by two-dimensional fluoroscopy. Using fluoroscopy may cause logistical difficulties during the surgery, especially by lengthening the operation time, jeopardizing the sterility of the surgical field and might expose the surgical team and the patient to ionizing radiation. Therefore, recently new methods for three-dimensional intraosseous navigation has been introduced, based on radiological registration, using plain x-ray imaging or computerized tomography [2]. These methods showed high navigation accuracy. In the most navigation systems the target accuracy of the distance is at most 1 mm, when targeting a single point with an angle of less than 1°. When considering the operator error, the accuracy of most systems is in the range of 2–4 mm for a targeted point and between 1° and 3° for a targeted trajectory[3]. However, despite the effectiveness of these navigation methods, they usually require sophisticated equipment and specially trained personnel, which are not always available for a broad clinical application. Therefore, a low cost and easily handled method for intraosseous surgical monitoring and guiding is desired. Sonographic monitoring might be an efficient method for this purpose. Previously we showed that by using a propagating ultrasonic beam through an aqueous milieu, which is directed as a jet into the drilled tract, a reliable measurement of a residual distance up to the opposite cortical boundary is possible, with an accuracy of 1 mm and with high agreement between sonographic and mechanical measurements [4]. This method is based on the sonographic resolution between trabecular surrounding of the drilled tract and cortical boundaries. This resolution is achieved because cortical bone has a constant matrix density of 2 gm/cm^3^, with a maximum porosity of 5–10% [5] and trabecular bone, which contains a significant proportion of open porous space filled with liquid bone marrow, has a lower density in the range of 0.2–0.7 gm/cm^3^ [5,6]. Therefore, we hypostasized that we could monitor the drilling direction in a vertebral pedicle by using sonographic measurements of the residual distances between the drilling tip inside the pedicle and the opposite bony cortex or sono-opaque bony boundaries.

## Materials and methods

To mimic the three-dimensional shape and the material properties of human vertebra we used the porcine L5 vertebra with the ratio of 0.2 between the trabecular and cortical bone densities (in human this ratio is very similar with value of 0.1) [7] and exact plastic replica of human L5 vertebra, i.e. the plastic replica was highly similar to the human vertebral shape and the ex vivo porcine specimen was representative for the material properties of human trabecular and cortical bone. Therefore, combined experiment on these two models should provide a good estimation of sonographic measurements expected in in human L5 vertebra, both regarding its shape and material properties.

**Fig 1:**
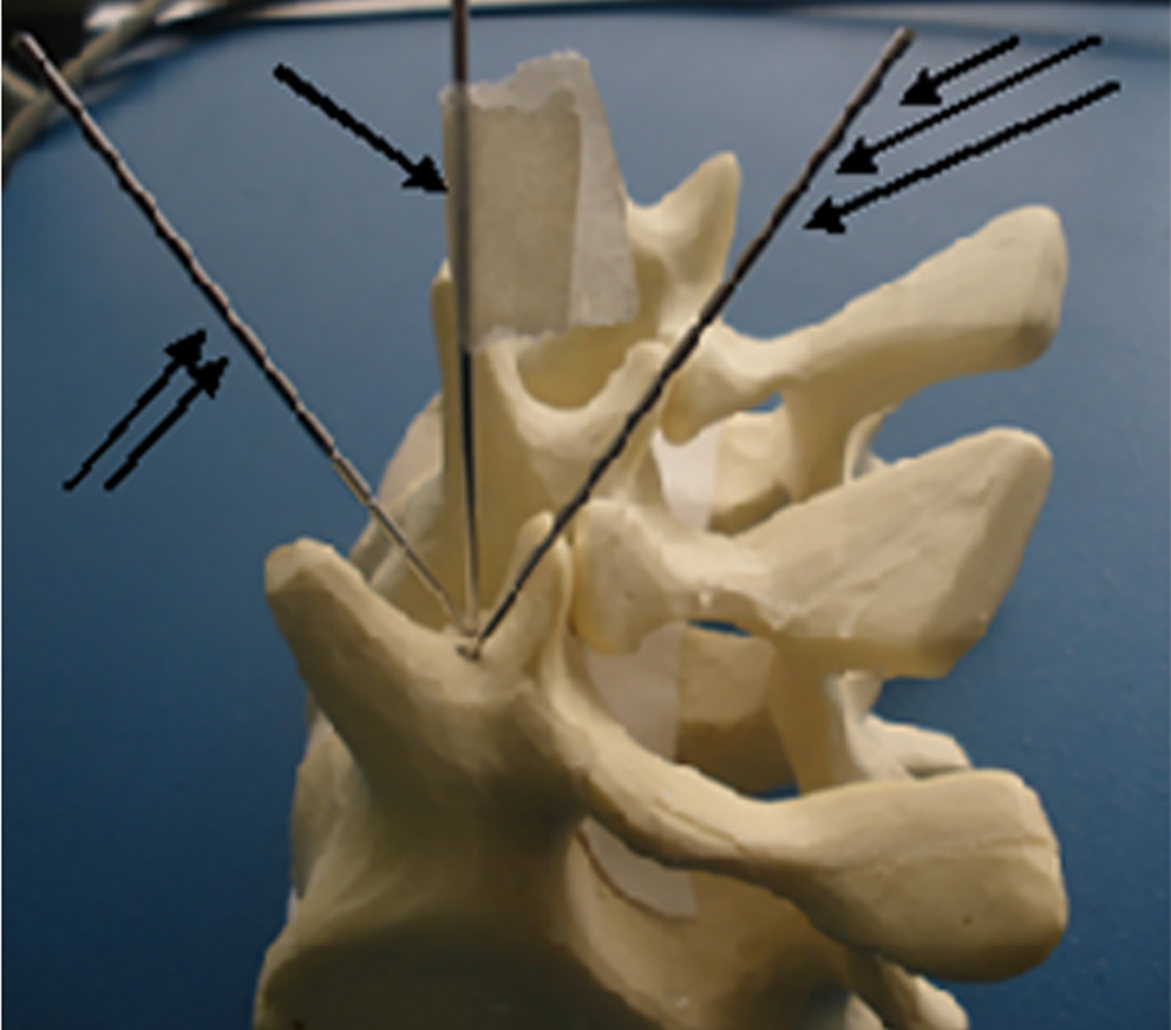
Demonstration of the generated by KW tracts in a vertebral pedicle (L5 vertebra plastic model). Central (desired route)—one arrow, medial and lateral (wrong routes)- two and three arrows accordingly.

The experiments were performed in two stages. Initially we used plastic models of human L5 vertebra (commercially obtained). For drilling we used a 2 mm in diameter metal Kirschner Wires (KW). The drilling route initiated from the posterior edge of the pedicle in the following directions: (a) central (desired), (b) medial (wrong) and (c) lateral (wrong) orientations in the pedicle mass (Fig 1).

**Fig 2:**
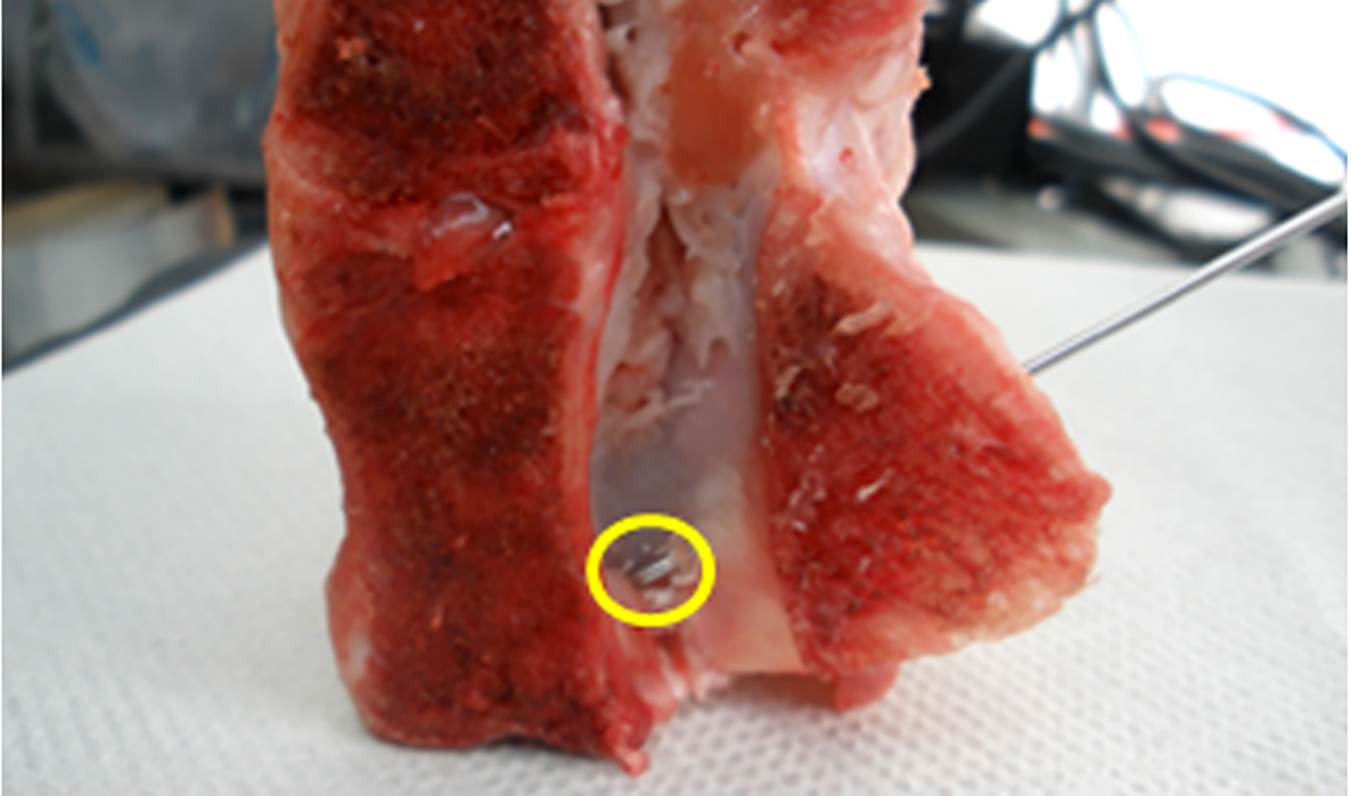
View on the sagittally divided L5 porcine lumbar vertebra specimen. A KW was drilled towards the medial direction until penetration of spinal canal (marked by a circle).

We measured the created drilled tracts’ (DT) lengths and the residual (to the opposite bony edge) distances (RD) mechanically (MM) and sonographically (US). The mechanical measurements were done by a caliper (precision of measurements of 0.1 mm), directly on the KW, and the residual distance was measured by an additional step of penetrating the opposite cortex by the same KW (Fig 2).

**Fig 3:**
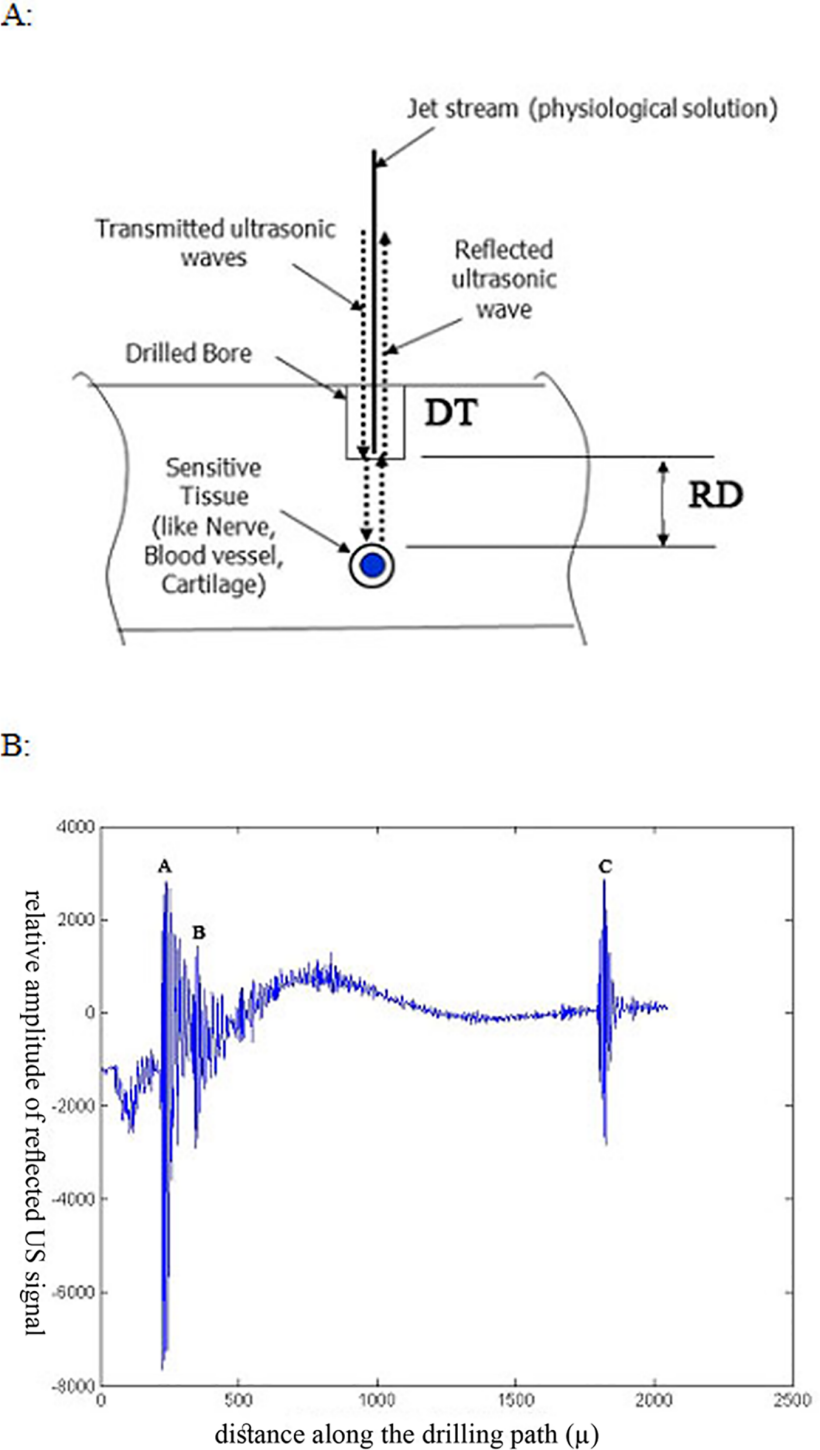
Intraosseous sonographic measurements. A: Schematic representation of the ultrasound wave propagation via water jet and its detection as a reflected wave. DT–drilled tract. RD–residual distance up to opposite cortex or dense trabecullar bone protecting vulnerable soft tissue structures. B: An example of a reflected sonographic pattern: A-B distance represents the DT, B-C distance represents the RD. The C point, which represents the reflection from the opposite dense bone, is detectable only up to 25–30 mm from the point of insertion.

**Fig 4:**
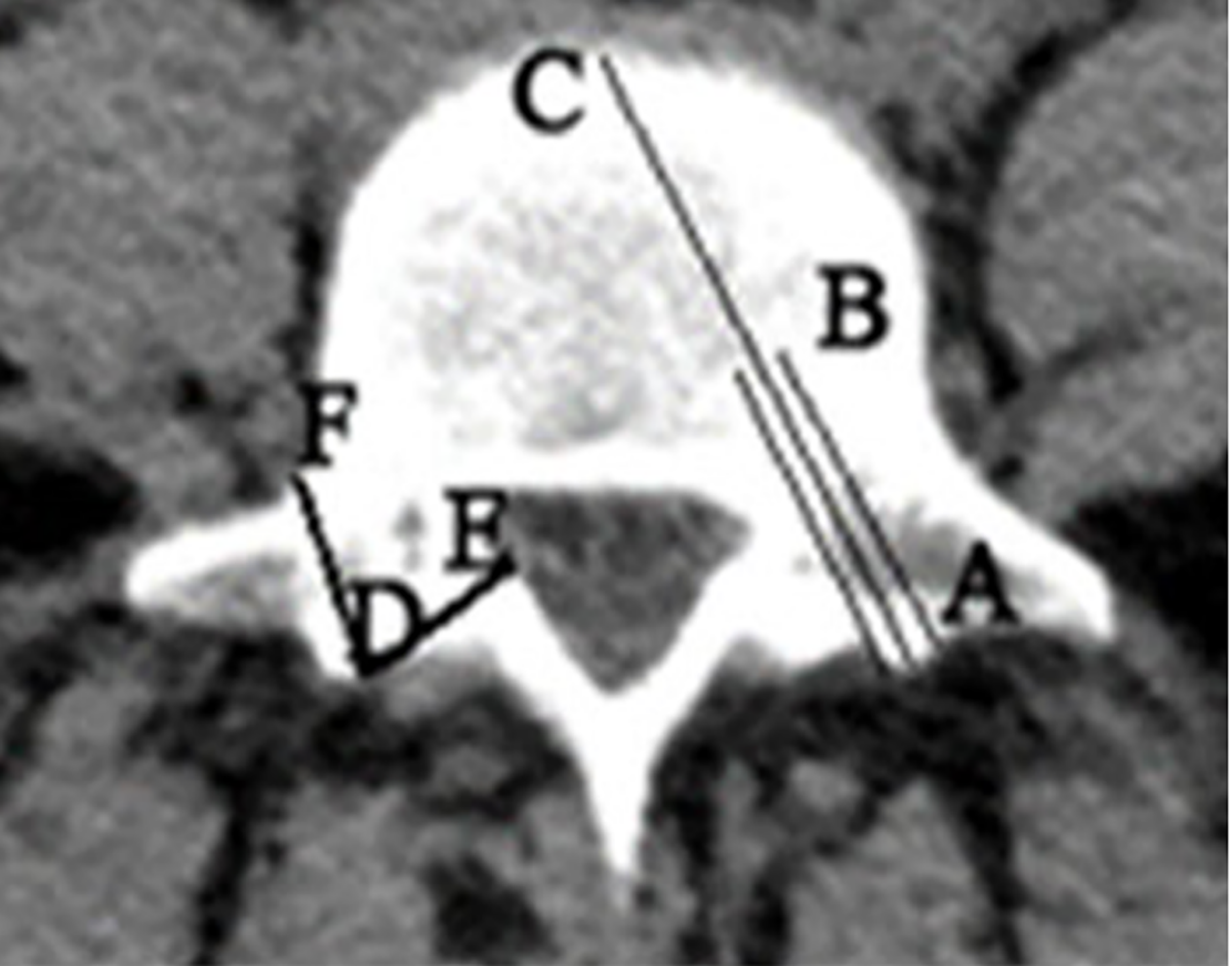
Schematic representation on a transverse CT image of human L5 vertebrae with indications of the drilling directions in lumbar vertebra pedicles. A-C is the desired direction of safe drilling. A-B is the drilled tract (DT), B-C is the residual distance (RD). D-E is a wrong medial drilling direction which jeopardizes the neural canal and its contents. D-F is a wrong lateral drilling direction which jeopardizes the neural root. D-E and D-F distances can be detected by the described sonographic method in order to be avoided.

The sonographic measurements were done by the specially designed ultrasonic device (JetGuide Ltd). This method utilizes measurements of reflected ultrasonic signals, i.e. the ultrasonic beam is directed into the drilled tract via a jet of water and the received back reflected ultrasonic signal, through the same pathway, is transformed to electronic signals, which are processed for measuring the distances between the bottom edge of the drilled tract and the opposite sono-opaque boundary (Fig 3A) [4]. Two different patterns of the detected US wave reflections are generated, i.e. low amplitude reflections from aqueous surrounding and highly reflected ultrasonic waves from the opposite boundary, which represents the bony cortex or outer sono-opaque boundary (Fig 3B). We measured the drilled tract (DT) and the residual distance (RD) to the opposite to the drilled edge sono-opaque boundary sonographically and mechanically (Fig 4).

The same method of drilling and measurements of the generated tracts and the residual distances were used on specimens of fresh porcine lumbar vertebra L5. The specimens were obtained from food industry source.

Currently the maximal distance measurement that is possible by this method is 25–30 mm. Therefore, sonographic measurements of the RD of centrally drilled tracts with the readings of “out of range” indicated that the distance is longer than 25 mm.

The measurements, mechanical and sonographic, were repeated six times by two different persons who were blinded regarding each other’s results. The individuals who performed the measurements were not involved in the statistical analysis.

**Fig 5:**
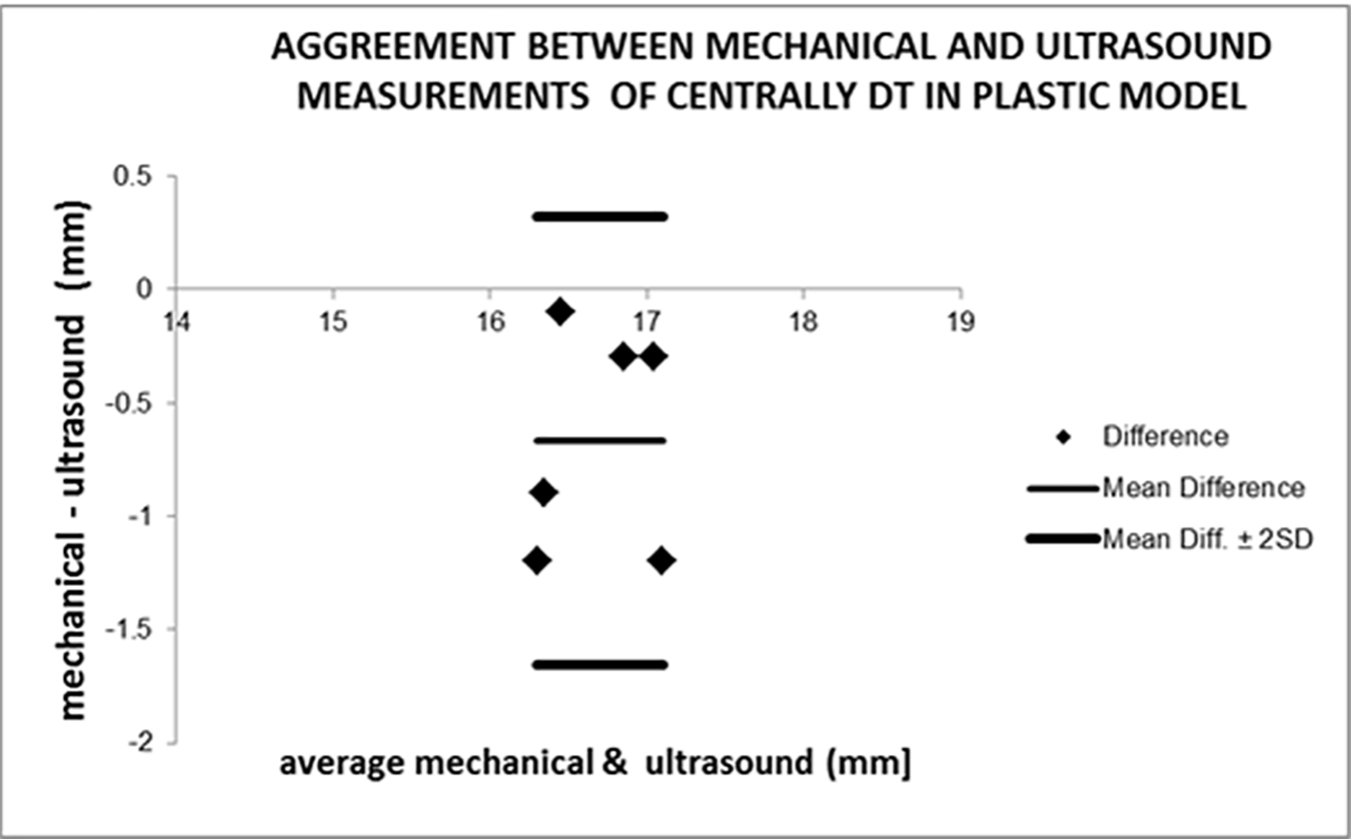
An example of Bland Altman plot for evaluation of the agreement between two measurement methods–sonographic and mechanical. DT–drilled tract in pedicle of L5 vertebra (plastic model).

We used the Bland Altman plotting [8] to estimate the agreement between the two methods of measurements of distances, i.e. MM and US (Fig 5), to evaluate the underestimation of US measurements, which might endanger the penetration of the opposite hard boundary or cortex. We have defined the "underestimation" value as equal to “(mean (MM-US) - 2SD)” per Bland Altman plot (Fig 5, Tables 1 and 2). The practical meaning of the “underestimation” is that a sonographic measurement of a distance to the opposite cortex is by mistake longer than the “true” mechanical measurement. Therefore, the underestimation by US measurements should have a negative value (below zero value).

**Table 1:** Data on agreement between sonographic (US) and mechanical measurements (MM) in plastic L5 vertebra model by Bland Altman plots. RD–residual distance, DT- drilled tract.

Direction of drilling	mean (MM–US) (mm)	2SD (mm)	(mean (MM-US) - 2SD) US underestimation (mm)
central DT	-066	0.98	-1.64
medial DT	-0.07	1.59	-1.66
medial RD	1.82	4.69	-2.87
lateral DT	0.37	3.42	-3.05
lateral RD	-0.50	0.49	-0.99

**Table 2:** Data on agreement between sonographic (US) and mechanical measurements (MM) in ex vivo L5 vertebra model by Bland Altman plots. RD–residual distance, DT- drilled tract.

Direction of drilling	mean (MM–US) (mm)	2SD (mm)	(mean (MM-US) - 2SD) US underestimation(mm)
medial DT	0.23	1.84	-1.61
medial RD	0.02	1.20	-1.18
lateral DT	-0.40	2.78	-3.18
lateral RD	2.91	4.23	-1.32

Because the Bland Altman’s plotting method might be internally biased [9], regression analysis was also used to consolidate the statistical evaluation of the results. Thus, the sonographic and mechanical measurements were analyzed by the Multiple Linear Regression method, after the normal distribution of values has been clarified [10]. The sonographic measurements were independent and mechanical measurements were dependent variables. We considered the p value below 0.05 as a significant for the comparison.

## Results

In the measurements on the plastic model the two methods had high agreement with maximal possible underestimation of US measurements in the central DT of 1.64 mm, in the medial DT—1.66 mm, in the medial RD—2.87 mm, in the lateral DT—3.05 mm and in the lateral RD—0.99 mm (Table 1).

In the measurements on the ex vivo model the two methods had high agreement with maximal possible underestimation of US measurements in the medial DT—1.61 mm, in the medial RD—1.18 mm, in the lateral DT—3.18 mm and in the lateral RD—1.32 mm (Table 2).

All the measurements, both in the phantom plastic model and in the ex-vivo experimental setups, showed that mechanical measurement can be predicted with high precision from sonographic measurement (Multiple Linear Regression: p<0.001, power of all tests equaled to 1.00; Tables 3 and 4). Per the results, for the prediction of the reference mechanical measurements the sonographically measured values should be corrected by 1 mm at the most.

**Table 3:** Mechanical measurements’ (MM) prediction of DT (drilled tract) length and residual distance (RD) length from ultrasonic (US) measurements in plastic model of L5 vertebra by Multiple Linear Regression analysis. RD–residual distance, DT- drilled tract.

Direction of drilling	mean US(mm) +/-SD	mean MM(mm)+/-SD	MM(US) mm =
central DT	16.35+/-0.46	17.01+/-0.41	-0.02 + 1.04*US
central RD	out of range	59.8+/-0.40	na
medial DT	9.23+/-0.69	10.03+/-0.31	0.07 + 1.08*US
medial RD	18.45-/-2.35	16.6+/-0.48	0.03 + 0.86*US
lateral DT	13.62+/-1.56	13.25+/-0.19	-0.15 + 0.97*US
lateral RD	25.25+/-017	26.00+/-0.52	0.04 + 1.02*US

**Table 4:** Mechanical measurements’ (MM) prediction of DT (drilled tract) length and residual distance (RD) length from ultrasonic (US) measurements in ex vivo specimen of L5 vertebra by Multiple Linear Regression analysis. RD–residual distance, DT- drilled tract.

Direction of drilling	mean US(mm) +/-SD	mean MM(mm)+/-SD	MM(US) mm
central RD	out of range	>40.00	na
medial DT	3.58+/-0.96	3.35+/-0.43	0.15 + 0.97*US
medial RD	5.65+/-0.35	5.60+/-1.19	0.04 + US
lateral DT	4.84+/-1.48	5.30+/-0.30	0.07 + 1.08*US
lateral RD	19.3+/-2.21	16.40+/-0.38	0.04 + 0.86*US

## Discussion

The rationale for this project is a further evaluation of the efficiency of a novel sonographic method for measurements of interosseous distances [4]. These measurements are very important in numerous orthopedic procedures, when hardware should be safely inserted into bone without penetrating the outer cortex. In a previous report, we showed in a relatively simple drilled structures, e.g. tubes or rectangles, in artificial models or in *ex vivo* specimens, the method of sonographic measurements is feasible and in high agreement with currently used radiographic imaging techniques [4]. In the current report, we tried to evaluate the sonographic distance measurements in a complex three-dimensional structure of lumbar vertebra, and particularly in its pedicle, which is a common target for drilling for insertion of different types of metal hardware for spinal stabilization or as a route for gaining access into a vertebral body. Although an experienced surgeon usually confident in the producing a correct direction of intra-pedicular drilling, a devastating mal-tracking can occur. Therefore for the safe drilling direction a reliable imaging and computerized navigation assistance is desirable[11]. We found that the correct "central direction" of the drilling can be detected by the sonographic measurements, when more than 25 mm of distance up to the opposite cortex is monitored, i.e. “out of range” measurements, in both phantom and *ex vivo* models.

If after starting the drilling the RD of 25 mm or less is detected, an alarming suspicion of the danger of the wrong route, either laterally or medially, of the drilling tract should be raised. This phenomenon was also observed in both phantom and *ex vivo* models. The wrong routes proximity to the opposite cortex, i.e. RD, can be predicted with precision of up to 1mm in comparison to mechanical measurements.

These data indicate that by using this innovative sonographic method of intraosseous measurement, precise enough for clinical use measurements are obtained, even in the complex three dimensional structures such as lumbar vertebra. Even without the corrections by the Multiple Linear Regression, a high agreement between the sonographic and mechanical measurements is apparent. According to the Bland Altman plotting the underestimation of sonographic measurements in comparison to mechanical measurements is of maximal value of 3.05 mm in the plastic model and 3.18 mm in the ex-vivo model.

From these data, we can safely claim that the presented sonographic measurement of distances from the drilled edge in the L5 vertebral pedicle up to the opposite bony boundary has a maximal underestimation error of 3.2 mm. This value of underestimation of sonographic measurements should be considered as acceptable in the clinical practice when it is taken into consideration in advance and is comparable to the sophisticated computerized navigation methods which are used clinically [2,6]. Therefore, by using this sonographic measurement a correct direction of drilling in the pedicle of lumbar L5 vertebra can be safely determined. This method should improve the safety of drilling in the lumbar vertebra during surgical procedures. Although this is an initial feasibility study, the presented data is highly indicative on the efficiency of the method for the future clinical use.

On this stage, we do not attempt to describe the precise surgical techniques for sonographic monitoring (those will be developed in the future according to the sonographic device design) but rather we intend to present the feasibility evidence for this method, which should be potentially readily available and precise.

We show that by using the sonographic measurements, a correct direction of drilling in the pedicle of L5 vertebra can be efficiently monitored and predicted with correction of 1–3 mm.
